# Supplementary material for: Physicochemical characteristics and high sensory acceptability in cappuccinos made with jackfruit seeds replacing cocoa powder
Source: PLoS One. 2018 Aug 15;13(8):e0197654. doi: 10.1371/journal.pone.0197654 (PMC6093646; doi:10.1371/journal.pone.0197654)
Supplement: S1 Table — I—Cappuccino with cocoa powder (Control); II—cappuccino base without cinnamon; III–cappuccino base without cocoa powder; IV-cappuccino base without coffee; V-cappuccino plus 10% dry jackfruit seed flour; VI- cappuccino plus 25%cocoa powder; VII-cappuccino with 15%cinnamon. (DOCX) [file pone.0197654.s001.docx]

| Ingredients (g) | Control | 50% | 75% | 100% | I | II | III | IV | V | VI | VII |
| --- | --- | --- | --- | --- | --- | --- | --- | --- | --- | --- | --- |
| Jackfruit seeds flour | 0.00 | 7.50 | 11.25 | 15.00 | 0.00 | 0.00 | 0.00 | 0.00 | 16.50 | 0.00 | 0.00 |
| Cocoa | 15.00 | 7.50 | 3.75 | 0.00 | 15.00 | 15.00 | 0.00 | 15.00 | 0.00 | 18.75 | 14.93 |
| Powdered sugar* | 41.50 | 41.50 | 41.50 | 41.50 | 41.50 | 41.50 | 41.50 | 41.50 | 41.03 | 39.94 | 41.31 |
| Powdered milk | 30.35 | 30.35 | 30.35 | 30.35 | 30.35 | 30.75 | 45.35 | 40.35 | 29.48 | 28.87 | 30.61 |
| Soluble coffee | 10.00 | 10.00 | 10.00 | 10.00 | 10.00 | 10.00 | 10.00 | 0.00 | 9.88 | 9.60 | 9.95 |
| Sodium bicarbonate | 1.75 | 1.75 | 1.75 | 1.75 | 1.75 | 1.75 | 1.75 | 1.75 | 1.73 | 1.66 | 1.74 |
| Cinnamon powder | 0.40 | 0.40 | 0.40 | 0.40 | 0.40 | 0.00 | 0.40 | 0.40 | 0.39 | 0.38 | 0.46 |
| Soy lecithin | 0.50 | 0.50 | 0.50 | 0.50 | 0.50 | 0.50 | 0.50 | 0.50 | 0.49 | 0.40 | 0.50 |
| Xanthan gum | 0.50 | 0.50 | 0.50 | 0.50 | 0.50 | 0.50 | 0.50 | 0.50 | 0.49 | 0.40 | 0.50 |
| TOTAL | 100 | 100 | 100 | 100 | 100 | 100 | 100 | 100 | 100 | 100 | 100 |

S1Table. Cappuccino formulations with 50%, 75% and 100% cocoa powder and formulations for QDA^®^ reference scale.
